# Crystal structure of (*E*)-3-(3,4-di­meth­oxy­phen­yl)-1-(1-hy­droxy­naphthalen-2-yl)prop-2-en-1-one

**DOI:** 10.1107/S2056989015008087

**Published:** 2015-04-30

**Authors:** K. S. Ezhilarasi, D. Reuben Jonathan, R. Vasanthi, B. K. Revathi, G. Usha

**Affiliations:** a PG and Research Department of Physics, Queen Mary’s College, Chennai-4, Tamilnadu, India; b PG and Research Department of Chemistry, Presidency College, Chennai-5, Tamil Nadu, India

**Keywords:** crystal structure, 1,3-diphenyl-2-propene-1-ones, chalcones, α,β-unsaturated carbonyl system, bifurcated C—H⋯O hydrogen bonds, O—H⋯O intra­molecular hydrogen bond

## Abstract

The mol­ecular structure of the title compound, C_21_H_18_O_4_, consists of a 3,4-di­meth­oxy­phenyl ring and a naphthalene ring system linked *via* a prop-2-en-1-one spacer. The mol­ecule is almost planar, with a dihedral angle between the benzene ring and the naphthalene ring system of 2.68 (12)°. There is an intra­molecular O—H⋯O hydrogen bond involving the adjacent hy­droxy and carbonyl groups. The mol­ecule has an *E* conformation about the C=C bond and the carbonyl group is *syn* with respect to the C=C bond. In the crystal, mol­ecules are linked by bifurcated C—H⋯(O,O) hydrogen bonds, enclosing an *R*_2_^1^(6) ring motif, and by a further C—H⋯O hydrogen bond, forming undulating sheets extending in *b-* and *c*-axis directions. There are π–π inter­actions between the sheets, involving inversion-related naphthalene and benzene rings [inter­centroid distance = 3.7452 (17) Å], forming a three-dimensional structure.

## Related literature   

For the biological activity of chalcone derivatives, see: Sashidhara *et al.* (2011 ▸); Go *et al.* (2005 ▸); Mukherjee *et al.* (2001 ▸); Liu *et al.* (2003 ▸); Sivakumar *et al.* (2007 ▸); Viana *et al.* (2003 ▸); Ducki *et al.* (1998 ▸); Rahman *et al.* (2007 ▸). For a related structure, see: Ahn *et al.* (2013 ▸). For the synthesis, see: Ezhilarasi *et al.* (2014 ▸); Sathya *et al.* (2014 ▸).
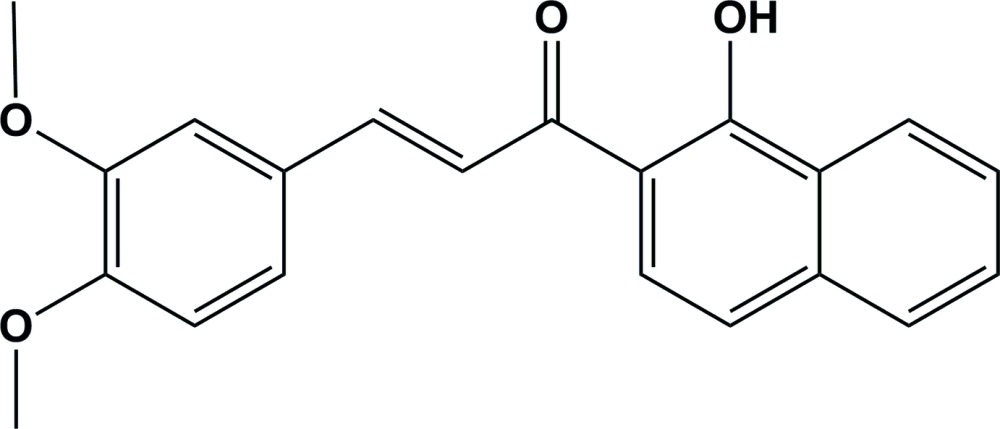


## Experimental   

### Crystal data  

C_21_H_18_O_4_
*M* *_r_* = 334.35
Monoclinic, 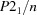

*a* = 9.8784 (10) Å
*b* = 15.4108 (15) Å
*c* = 11.2288 (11) Å
β = 92.674 (2)°
*V* = 1707.5 (3) Å^3^
*Z* = 4
Mo *K*α radiation
μ = 0.09 mm^−1^
*T* = 293 K
0.30 × 0.25 × 0.20 mm

### Data collection  

Bruker Kappa APEXII CCD diffractometer
Absorption correction: multi-scan (*SADABS*; Bruker, 2004 ▸) *T*_min_ = 0.973, *T*_max_ = 0.982
34671 measured reflections
3568 independent reflections
1992 reflections with *I* > 2σ(*I*)
*R*_int_ = 0.051

### Refinement  

*R*[*F* ^2^ > 2σ(*F* ^2^)] = 0.068
*wR*(*F* ^2^) = 0.247
*S* = 1.08
3568 reflections
230 parameters
H-atom parameters constrained
Δρ_max_ = 0.29 e Å^−3^
Δρ_min_ = −0.29 e Å^−3^

### 

Data collection: *APEX2* (Bruker, 2004 ▸); cell refinement: *APEX2* and *SAINT* (Bruker, 2004 ▸); data reduction: *SAINT* and *XPREP* (Bruker, 2004 ▸); program(s) used to solve structure: *SHELXS97* (Sheldrick, 2008 ▸); program(s) used to refine structure: *SHELXL2014* (Sheldrick, 2015 ▸); molecular graphics: *ORTEP-3 for Windows* (Farrugia, 2012 ▸); software used to prepare material for publication: *SHELXL2014* and *PLATON* (Spek, 2009 ▸).

## Supplementary Material

Crystal structure: contains datablock(s) I, Global. DOI: 10.1107/S2056989015008087/su5109sup1.cif

Structure factors: contains datablock(s) I. DOI: 10.1107/S2056989015008087/su5109Isup2.hkl

Supporting information file. DOI: 10.1107/S2056989015008087/su5109Isup3.cml

**Fig. 1.**
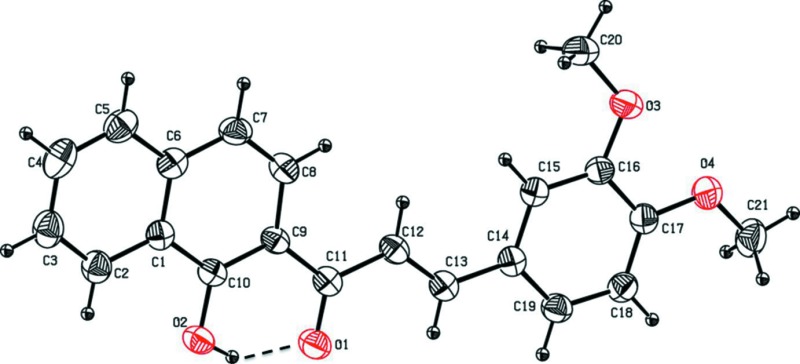
The molecular structure of the title compound, with atom labelling. Displacement ellipsoids are drawn at the 30% probability level.

**Fig. 2.**
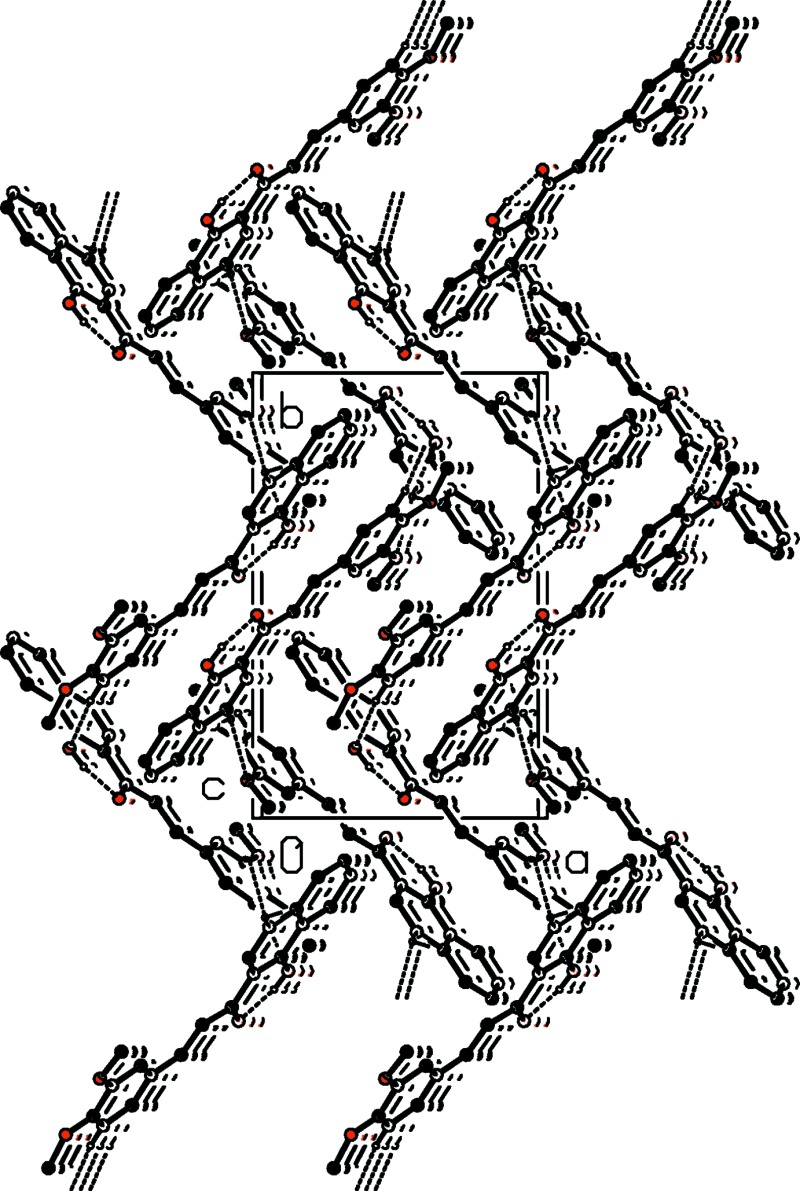
A view along the c axis of the crystal packing of the title compound. The hydrogen bonds are shown as dashed lines (see Table 1 for details).

. DOI: 10.1107/S2056989015008087/su5109fig1.tif
The mol­ecular structure of the title compound, with atom labelling. Displacement ellipsoids are drawn at the 30% probability level.

c . DOI: 10.1107/S2056989015008087/su5109fig2.tif
A view along the *c* axis of the crystal packing of the title compound. The hydrogen bonds are shown as dashed lines (see Table 1 for details).

CCDC reference: 1051679

Additional supporting information:  crystallographic information; 3D view; checkCIF report

## Figures and Tables

**Table 1:** Hydrogen-bond geometry (, )

*D*H*A*	*D*H	H*A*	*D* *A*	*D*H*A*
O2H2*A*O1	0.82	1.75	2.482(3)	148
C7H7O3^i^	0.93	2.57	3.368(4)	144
C7H7O4^i^	0.93	2.59	3.445(4)	152
C18H18O2^ii^	0.93	2.43	3.333(4)	165

Symmetry codes: (i) 
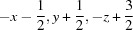
; (ii) 
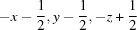
.

## supplementary crystallographic information

### S1. Synthesis and crystallization

The title compound was synthesized following the reported procedures (Ezhilarasi *et al.*, 2014; Sathya *et al.*, 2014).

### S2. Refinement

Crystal data, data collection and structure refinement details are summarized in Table 2. H atoms were positioned geometrically and treated as riding atoms: C—H = 0.93–0.96 Å, O—H = 0.82 Å with *U*_iso_(H)= 1.5*U*_eq_(C) for methyl H atoms and 1.2Ueq(O,C) for other H atoms.

### S3. Comment

Chalcones are 1,3-di­phenyl-2-propene-1-ones, in which two aromatic rings are linked by a three carbon α,β-unsaturated carbonyl system.These are abundant in edible plants and are considered to be precursors of flavonoids and isoflavonoids (Rahman *et al.*, 2007). The presence of the enone functionality in the chalcone moiety confers biological activity upon it, such as anti­inflammatory (Sashidhara *et al.*, 2011), anti­fungal (Go *et al.*, 2005) anti­oxidant (Mukherjee *et al.*, 2001), anti­malarial(Liu *et al.*, 2003) anti­tuberculosis (Sivakumar *et al.*, 2007), analgesic (Viana *et al.*, 2003], and anti­tumor (Ducki *et al.*, 1998) activities. As part of our attempts to investigate the substituent effects of chalcones on molecular structures and hence activities, the title compound was synthesized and its crystal structure was determined.

The molecular structure of the title compound is shown in Fig. 1. The bond distances and angles are similar to those reported for a similar structure (Ahn *et al.*, 2013). The molecule is almost planar with a dihedral angle between the benzene and naphthalene rings of 2.65 (1)°. The torsion angles of C20—O3—C16—C17 = 171.3 (3)° and C21—O4—C17—C16 = -174.0 (3)°, indicates the *trans* orientation of the two meth­oxy groups.

In the crystal, molecules are linked by bifurcated C—H···(O,O) hydrogen bonds, enclosing an *R*_2_^1^(6) ring motif, and a further C—H···O hydrogen bond forming undulating sheets extending in the *b* and *c* directions. (Fig. 2 and Table 1). There are π–π inter­actions between the sheets, involving inversion related naphthalene and benzene rings, resulting in a three-dimensional structure [Cg2···Cg3^i^ = 3.7452 (17) Å; Cg2 and Cg3 are the centroids or rings and C14—C19; symmetry code: (i) -x, -y+1, -z+1].

### Crystal data

**Table:**

C_21_H_18_O_4_	*F*(000) = 704
*M**_r_* = 334.35	*D*_x_ = 1.301 Mg m^−^^3^
Monoclinic, *P*2_1_/*n*	Mo *K*α radiation, λ = 0.71073 Å
*a* = 9.8784 (10) Å	Cell parameters from 3582 reflections
*b* = 15.4108 (15) Å	θ = 2.3–26.6°
*c* = 11.2288 (11) Å	µ = 0.09 mm^−^^1^
β = 92.674 (2)°	*T* = 293 K
*V* = 1707.5 (3) Å^3^	Block, redish
*Z* = 4	0.30 × 0.25 × 0.20 mm

### Data collection

**Table:**

Bruker Kappa APEXII CCD diffractometer	3568 independent reflections
Radiation source: fine-focus sealed tube	1992 reflections with *I* > 2σ(*I*)
Graphite monochromator	*R*_int_ = 0.051
ω and φ scan	θ_max_ = 26.6°, θ_min_ = 2.3°
Absorption correction: multi-scan (*SADABS*; Bruker, 2004)	*h* = −12→12
*T*_min_ = 0.973, *T*_max_ = 0.982	*k* = −19→19
34671 measured reflections	*l* = −14→14

### Refinement

**Table:**

Refinement on *F*^2^	Secondary atom site location: difference Fourier map
Least-squares matrix: full	Hydrogen site location: inferred from neighbouring sites
*R*[*F*^2^ > 2σ(*F*^2^)] = 0.068	H-atom parameters constrained
*wR*(*F*^2^) = 0.247	*w* = 1/[σ^2^(*F*_o_^2^) + (0.125*P*)^2^ + 0.6413*P*] where *P* = (*F*_o_^2^ + 2*F*_c_^2^)/3
*S* = 1.08	(Δ/σ)_max_ < 0.001
3568 reflections	Δρ_max_ = 0.29 e Å^−^^3^
230 parameters	Δρ_min_ = −0.29 e Å^−^^3^
0 restraints	Extinction correction: *SHELXL2014* (Sheldrick, 2015), Fc^*^=kFc[1+0.001xFc^2^λ^3^/sin(2θ)]^-1/4^
Primary atom site location: structure-invariant direct methods	Extinction coefficient: 0.212 (16)

### Special details

**Table:**

Geometry. All esds (except the esd in the dihedral angle between two l.s. planes) are estimated using the full covariance matrix. The cell esds are taken into account individually in the estimation of esds in distances, angles and torsion angles; correlations between esds in cell parameters are only used when they are defined by crystal symmetry. An approximate (isotropic) treatment of cell esds is used for estimating esds involving l.s. planes.

### Fractional atomic coordinates and isotropic or equivalent isotropic displacement parameters (Å^2^)

**Table:**

	*x*	*y*	*z*	*U*_iso_*/*U*_eq_	
O1	−0.0402 (2)	0.54423 (14)	0.26187 (18)	0.0626 (6)	
O2	0.1324 (2)	0.65677 (13)	0.22420 (16)	0.0568 (6)	
H2A	0.0852	0.6136	0.2119	0.085*	
O3	−0.5102 (2)	0.41489 (16)	0.74635 (18)	0.0691 (7)	
O4	−0.6337 (2)	0.28908 (15)	0.63862 (19)	0.0664 (7)	
C1	0.1882 (3)	0.76195 (17)	0.3705 (2)	0.0468 (7)	
C2	0.2833 (3)	0.8011 (2)	0.2974 (3)	0.0589 (8)	
H2	0.2972	0.7784	0.2222	0.071*	
C3	0.3548 (3)	0.8720 (2)	0.3366 (3)	0.0702 (10)	
H3	0.4157	0.8983	0.2872	0.084*	
C4	0.3372 (4)	0.9050 (2)	0.4501 (4)	0.0757 (10)	
H4	0.3872	0.9530	0.4762	0.091*	
C5	0.2474 (3)	0.8680 (2)	0.5235 (3)	0.0667 (9)	
H5	0.2372	0.8907	0.5993	0.080*	
C6	0.1701 (3)	0.79568 (19)	0.4855 (3)	0.0533 (8)	
C7	0.0772 (3)	0.7555 (2)	0.5598 (3)	0.0599 (8)	
H7	0.0658	0.7770	0.6361	0.072*	
C8	0.0047 (3)	0.68597 (19)	0.5208 (3)	0.0552 (8)	
H8	−0.0557	0.6606	0.5715	0.066*	
C9	0.0170 (3)	0.65037 (17)	0.4059 (2)	0.0439 (7)	
C10	0.1113 (3)	0.68788 (17)	0.3329 (2)	0.0445 (7)	
C11	−0.0608 (3)	0.57509 (17)	0.3629 (2)	0.0479 (7)	
C12	−0.1620 (3)	0.53573 (18)	0.4362 (3)	0.0505 (7)	
H12	−0.1748	0.5582	0.5118	0.061*	
C13	−0.2369 (3)	0.46840 (19)	0.3981 (3)	0.0514 (7)	
H13	−0.2211	0.4483	0.3219	0.062*	
C14	−0.3399 (3)	0.42287 (18)	0.4610 (2)	0.0484 (7)	
C15	−0.3725 (3)	0.44398 (18)	0.5770 (2)	0.0482 (7)	
H15	−0.3268	0.4891	0.6165	0.058*	
C16	−0.4709 (3)	0.39945 (19)	0.6339 (2)	0.0494 (7)	
C17	−0.5390 (3)	0.33076 (19)	0.5753 (3)	0.0502 (7)	
C18	−0.5085 (3)	0.30938 (19)	0.4609 (3)	0.0549 (8)	
H18	−0.5543	0.2644	0.4213	0.066*	
C19	−0.4089 (3)	0.35528 (19)	0.4049 (3)	0.0565 (8)	
H19	−0.3881	0.3402	0.3277	0.068*	
C20	−0.4327 (4)	0.4750 (3)	0.8165 (3)	0.0800 (11)	
H20A	−0.4646	0.4758	0.8960	0.120*	
H20B	−0.4418	0.5318	0.7820	0.120*	
H20C	−0.3391	0.4580	0.8191	0.120*	
C21	−0.6958 (4)	0.2140 (2)	0.5876 (3)	0.0770 (10)	
H21A	−0.7562	0.1892	0.6429	0.116*	
H21B	−0.6273	0.1723	0.5700	0.116*	
H21C	−0.7459	0.2295	0.5155	0.116*	

### Atomic displacement parameters (Å^2^)

**Table:**

	*U*^11^	*U*^22^	*U*^33^	*U*^12^	*U*^13^	*U*^23^
O1	0.0739 (15)	0.0598 (13)	0.0553 (13)	−0.0084 (11)	0.0161 (10)	−0.0062 (10)
O2	0.0683 (14)	0.0591 (13)	0.0440 (11)	−0.0023 (10)	0.0119 (10)	0.0030 (9)
O3	0.0801 (15)	0.0796 (16)	0.0486 (13)	−0.0189 (12)	0.0110 (11)	−0.0048 (10)
O4	0.0678 (14)	0.0696 (15)	0.0620 (13)	−0.0185 (11)	0.0060 (11)	0.0058 (11)
C1	0.0443 (15)	0.0449 (15)	0.0511 (16)	0.0067 (12)	−0.0003 (12)	0.0070 (12)
C2	0.0584 (19)	0.0583 (18)	0.0597 (18)	−0.0032 (15)	0.0013 (15)	0.0102 (14)
C3	0.064 (2)	0.065 (2)	0.081 (2)	−0.0109 (17)	0.0006 (18)	0.0163 (18)
C4	0.067 (2)	0.058 (2)	0.101 (3)	−0.0055 (17)	−0.016 (2)	−0.0005 (19)
C5	0.065 (2)	0.058 (2)	0.076 (2)	0.0049 (16)	−0.0085 (17)	−0.0108 (16)
C6	0.0528 (17)	0.0486 (16)	0.0580 (17)	0.0084 (13)	−0.0023 (14)	−0.0008 (13)
C7	0.0643 (19)	0.065 (2)	0.0507 (17)	0.0067 (16)	0.0061 (14)	−0.0116 (15)
C8	0.0566 (18)	0.0566 (18)	0.0536 (17)	0.0026 (14)	0.0134 (14)	0.0002 (14)
C9	0.0465 (15)	0.0433 (15)	0.0424 (14)	0.0090 (11)	0.0068 (12)	0.0019 (11)
C10	0.0476 (16)	0.0442 (15)	0.0417 (15)	0.0094 (12)	0.0031 (12)	0.0041 (11)
C11	0.0500 (16)	0.0440 (15)	0.0499 (16)	0.0081 (12)	0.0056 (12)	0.0051 (12)
C12	0.0536 (17)	0.0500 (16)	0.0485 (16)	0.0039 (13)	0.0094 (13)	0.0060 (12)
C13	0.0510 (17)	0.0515 (17)	0.0519 (16)	0.0057 (13)	0.0053 (13)	0.0049 (13)
C14	0.0455 (15)	0.0469 (16)	0.0529 (17)	0.0042 (12)	0.0027 (12)	0.0064 (12)
C15	0.0475 (16)	0.0463 (15)	0.0506 (16)	0.0017 (12)	0.0001 (12)	0.0034 (12)
C16	0.0506 (16)	0.0539 (17)	0.0435 (15)	0.0011 (13)	−0.0004 (12)	0.0036 (12)
C17	0.0481 (16)	0.0517 (16)	0.0507 (17)	0.0012 (13)	0.0007 (13)	0.0094 (13)
C18	0.0562 (18)	0.0526 (17)	0.0554 (18)	−0.0042 (14)	−0.0023 (14)	−0.0007 (13)
C19	0.0605 (18)	0.0581 (18)	0.0512 (17)	0.0002 (14)	0.0064 (14)	−0.0053 (14)
C20	0.093 (3)	0.092 (3)	0.056 (2)	−0.018 (2)	0.0084 (18)	−0.0162 (18)
C21	0.069 (2)	0.073 (2)	0.089 (3)	−0.0219 (18)	0.0025 (19)	0.0060 (19)

### Geometric parameters (Å, º)

**Table:**

O1—C11	1.255 (3)	C9—C10	1.395 (4)
O2—C10	1.336 (3)	C9—C11	1.462 (4)
O2—H2A	0.8200	C11—C12	1.457 (4)
O3—C16	1.359 (3)	C12—C13	1.333 (4)
O3—C20	1.417 (4)	C12—H12	0.9300
O4—C17	1.361 (3)	C13—C14	1.446 (4)
O4—C21	1.418 (4)	C13—H13	0.9300
C1—C6	1.411 (4)	C14—C19	1.381 (4)
C1—C2	1.411 (4)	C14—C15	1.395 (4)
C1—C10	1.425 (4)	C15—C16	1.372 (4)
C2—C3	1.363 (5)	C15—H15	0.9300
C2—H2	0.9300	C16—C17	1.402 (4)
C3—C4	1.391 (5)	C17—C18	1.374 (4)
C3—H3	0.9300	C18—C19	1.385 (4)
C4—C5	1.365 (5)	C18—H18	0.9300
C4—H4	0.9300	C19—H19	0.9300
C5—C6	1.406 (4)	C20—H20A	0.9600
C5—H5	0.9300	C20—H20B	0.9600
C6—C7	1.412 (4)	C20—H20C	0.9600
C7—C8	1.350 (4)	C21—H21A	0.9600
C7—H7	0.9300	C21—H21B	0.9600
C8—C9	1.412 (4)	C21—H21C	0.9600
C8—H8	0.9300		
			
C10—O2—H2A	109.5	C13—C12—C11	121.8 (3)
C16—O3—C20	117.4 (2)	C13—C12—H12	119.1
C17—O4—C21	118.0 (2)	C11—C12—H12	119.1
C6—C1—C2	119.4 (3)	C12—C13—C14	127.8 (3)
C6—C1—C10	118.6 (2)	C12—C13—H13	116.1
C2—C1—C10	122.0 (3)	C14—C13—H13	116.1
C3—C2—C1	120.2 (3)	C19—C14—C15	118.1 (3)
C3—C2—H2	119.9	C19—C14—C13	119.2 (3)
C1—C2—H2	119.9	C15—C14—C13	122.8 (3)
C2—C3—C4	120.3 (3)	C16—C15—C14	121.2 (3)
C2—C3—H3	119.9	C16—C15—H15	119.4
C4—C3—H3	119.9	C14—C15—H15	119.4
C5—C4—C3	120.9 (3)	O3—C16—C15	125.8 (3)
C5—C4—H4	119.6	O3—C16—C17	114.6 (2)
C3—C4—H4	119.6	C15—C16—C17	119.6 (3)
C4—C5—C6	120.5 (3)	O4—C17—C18	124.1 (3)
C4—C5—H5	119.8	O4—C17—C16	116.0 (3)
C6—C5—H5	119.8	C18—C17—C16	119.9 (3)
C5—C6—C1	118.7 (3)	C17—C18—C19	119.6 (3)
C5—C6—C7	121.8 (3)	C17—C18—H18	120.2
C1—C6—C7	119.5 (3)	C19—C18—H18	120.2
C8—C7—C6	120.4 (3)	C14—C19—C18	121.6 (3)
C8—C7—H7	119.8	C14—C19—H19	119.2
C6—C7—H7	119.8	C18—C19—H19	119.2
C7—C8—C9	122.5 (3)	O3—C20—H20A	109.5
C7—C8—H8	118.7	O3—C20—H20B	109.5
C9—C8—H8	118.7	H20A—C20—H20B	109.5
C10—C9—C8	117.7 (3)	O3—C20—H20C	109.5
C10—C9—C11	119.3 (2)	H20A—C20—H20C	109.5
C8—C9—C11	122.9 (2)	H20B—C20—H20C	109.5
O2—C10—C9	121.7 (2)	O4—C21—H21A	109.5
O2—C10—C1	117.0 (2)	O4—C21—H21B	109.5
C9—C10—C1	121.3 (2)	H21A—C21—H21B	109.5
O1—C11—C12	119.8 (3)	O4—C21—H21C	109.5
O1—C11—C9	119.6 (2)	H21A—C21—H21C	109.5
C12—C11—C9	120.5 (2)	H21B—C21—H21C	109.5
			
C6—C1—C2—C3	−1.4 (4)	C8—C9—C11—O1	−176.6 (3)
C10—C1—C2—C3	179.9 (3)	C10—C9—C11—C12	−178.7 (2)
C1—C2—C3—C4	1.6 (5)	C8—C9—C11—C12	3.4 (4)
C2—C3—C4—C5	−0.7 (5)	O1—C11—C12—C13	−1.7 (4)
C3—C4—C5—C6	−0.4 (5)	C9—C11—C12—C13	178.3 (2)
C4—C5—C6—C1	0.5 (4)	C11—C12—C13—C14	179.7 (3)
C4—C5—C6—C7	179.5 (3)	C12—C13—C14—C19	178.6 (3)
C2—C1—C6—C5	0.4 (4)	C12—C13—C14—C15	−1.7 (5)
C10—C1—C6—C5	179.1 (3)	C19—C14—C15—C16	−0.4 (4)
C2—C1—C6—C7	−178.6 (3)	C13—C14—C15—C16	179.9 (2)
C10—C1—C6—C7	0.1 (4)	C20—O3—C16—C15	−8.0 (4)
C5—C6—C7—C8	−179.8 (3)	C20—O3—C16—C17	171.3 (3)
C1—C6—C7—C8	−0.9 (5)	C14—C15—C16—O3	−180.0 (3)
C6—C7—C8—C9	−0.2 (5)	C14—C15—C16—C17	0.8 (4)
C7—C8—C9—C10	2.0 (4)	C21—O4—C17—C18	6.1 (4)
C7—C8—C9—C11	179.9 (3)	C21—O4—C17—C16	−174.0 (3)
C8—C9—C10—O2	178.1 (2)	O3—C16—C17—O4	−0.3 (4)
C11—C9—C10—O2	0.0 (4)	C15—C16—C17—O4	179.0 (2)
C8—C9—C10—C1	−2.7 (4)	O3—C16—C17—C18	179.6 (3)
C11—C9—C10—C1	179.2 (2)	C15—C16—C17—C18	−1.0 (4)
C6—C1—C10—O2	−179.0 (2)	O4—C17—C18—C19	−179.1 (3)
C2—C1—C10—O2	−0.3 (4)	C16—C17—C18—C19	0.9 (4)
C6—C1—C10—C9	1.8 (4)	C15—C14—C19—C18	0.3 (4)
C2—C1—C10—C9	−179.6 (2)	C13—C14—C19—C18	180.0 (3)
C10—C9—C11—O1	1.3 (4)	C17—C18—C19—C14	−0.6 (5)

### Hydrogen-bond geometry (Å, º)

**Table:**

*D*—H···*A*	*D*—H	H···*A*	*D*···*A*	*D*—H···*A*
O2—H2*A*···O1	0.82	1.75	2.482 (3)	148
C7—H7···O3^i^	0.93	2.57	3.368 (4)	144
C7—H7···O4^i^	0.93	2.59	3.445 (4)	152
C18—H18···O2^ii^	0.93	2.43	3.333 (4)	165

Symmetry codes: (i) −*x*−1/2, *y*+1/2, −*z*+3/2; (ii) −*x*−1/2, *y*−1/2, −*z*+1/2.
