# Supplementary figures and images for: Crystal structure of (E)-3-(3,4-di­meth­oxy­phen­yl)-1-(1-hy­droxy­naphthalen-2-yl)prop-2-en-1-one
Source: Acta Crystallogr E Crystallogr Commun. 2015 Apr 30;71(Pt 5):o371–2. doi: 10.1107/S2056989015008087 (PMC4420054; doi:10.1107/S2056989015008087)

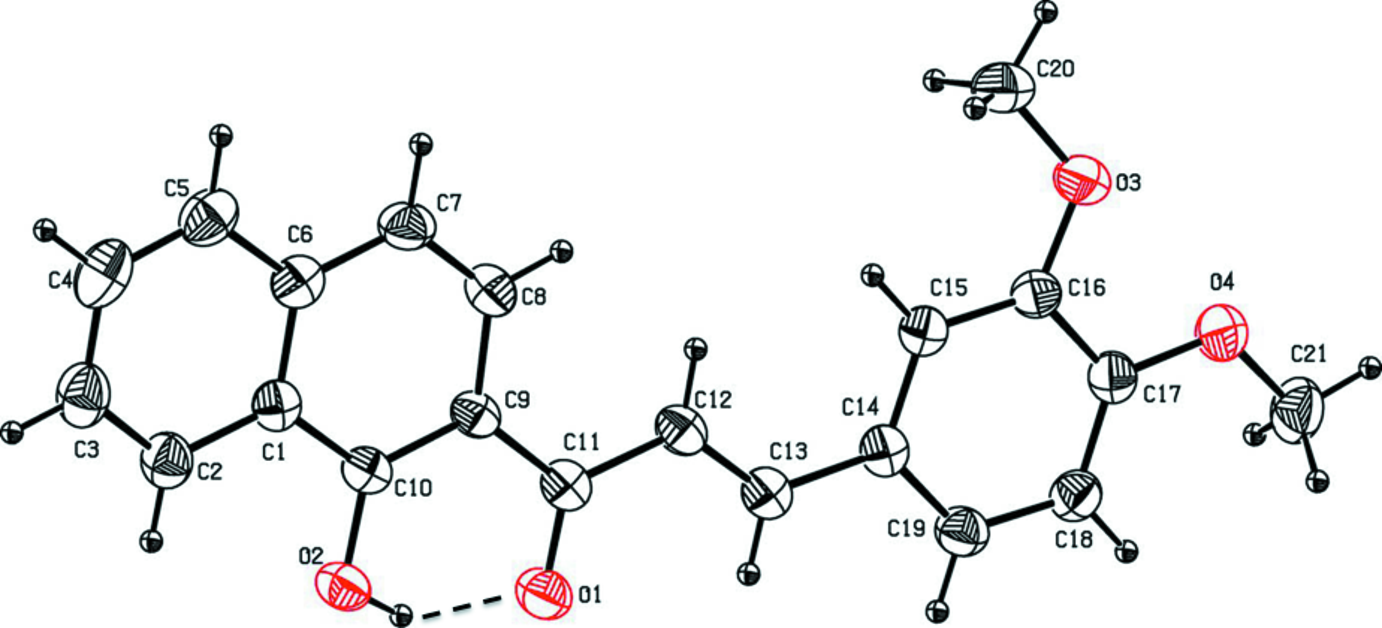

Supplement: Supplementary file 4 [file e-71-0o371-fig1.tif]

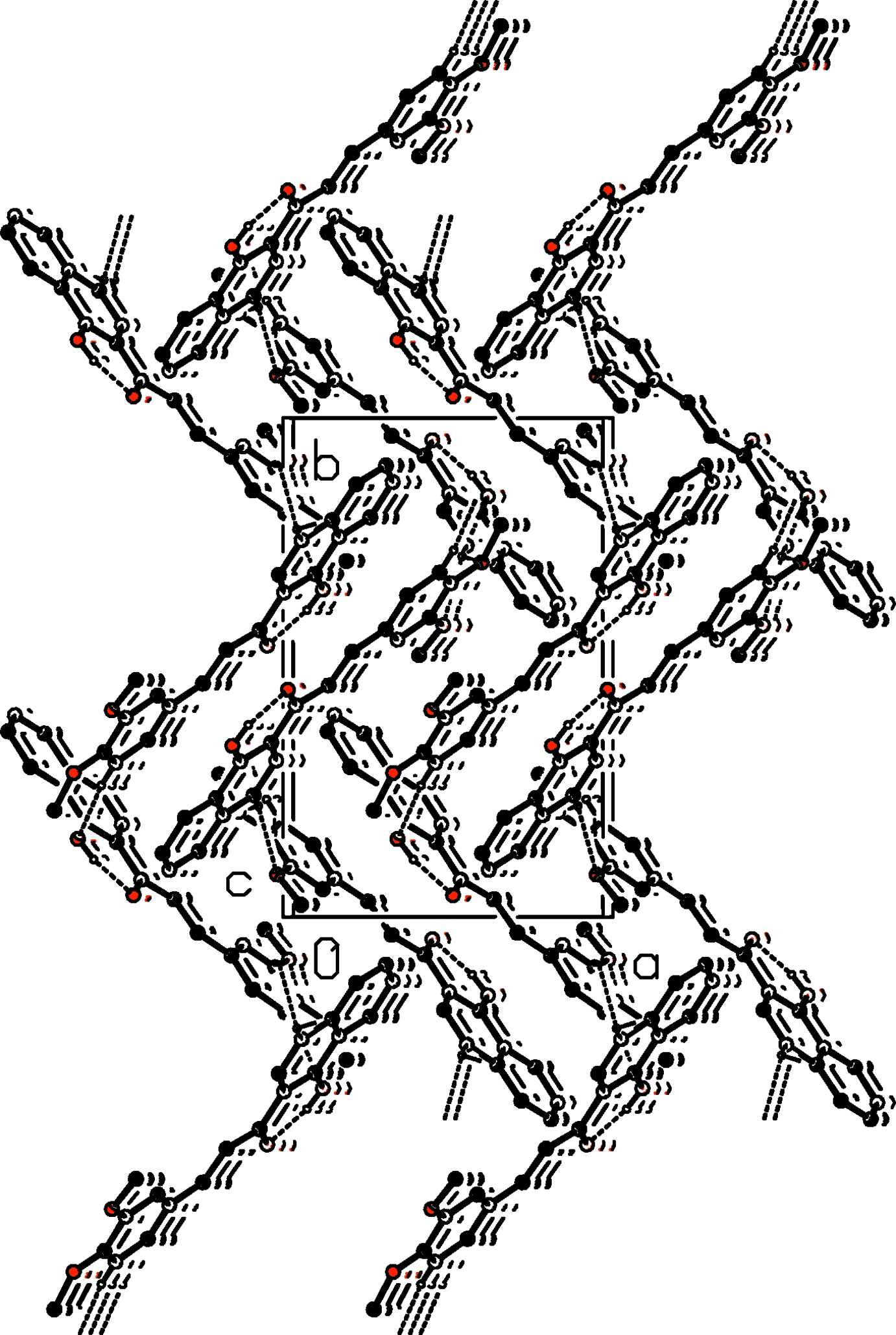

Supplement: Supplementary file 5 [file e-71-0o371-fig2.tif]
